# Research on Vertical Professional Collaborative Evaluation Tools of Healthcare System Based on the Tight County Healthcare Alliance in China

**DOI:** 10.5334/ijic.8603

**Published:** 2025-11-11

**Authors:** Ying Zheng, Li Li, Jia Hu

**Affiliations:** 1 Center for Health Policy and Management, Institute of Medical Information & Library, Chinese Academy of Medical Sciences & Peking Union Medical College, Beijing 100020, CN

**Keywords:** integrated healthcare system, vertical professional integration, evaluation tools, County, China

## Abstract

**Objectives:**

To develop the vertical professional collaborative evaluation tools to promote the establishment of integrated healthcare system in China.

**Method:**

Based on the previous theoretical framework, the evaluation system was developed and 450 doctors and other health professionals in tight county healthcare alliance in D county of H province were selected and interviewed. Through stratified cluster equal proportion random sampling method with an effective recovery rate of 93.33%, reliability and validity were tested with exploratory factor analysis, Cronbach’s α and structural equation model method.

**Results:**

The cumulative contribution rate of the five common factors was 72.23%, the Cronbach’s α of whole is 0.846. Except for the common factor F4, the Cronbach’s α of other common factors were greater than 0.7. The component reliability (CR) of 5 common factors were all greater than 0.7 and the average coefficients of variation extraction (AVE) were all greater than 0.6. In the revised model (M1), the P values of the standard regression coefficients of F1, F2, F3, F4 and those of the corresponding items and factors were all smaller than 0.05, and the model fitting indexes of were all better than those of the initial model (M0).

**Conclusions:**

The vertical professional collaborative evaluation tools of healthcare system constructed in this paper contain 4 dimensions: (1) Value compatibility and trust, defined as the alignment of health-related values, cultural norms, and behavioral expectations across different provider levels (e.g., primary vs. tertiary care) and specialties (e.g., physicians vs. nurses), operationalized through shared decision-making and perceived reliability; (2) Communication and coordination mechanisms, encompassing systems for bidirectional information flow (e.g., standardized referral protocols, interoperable IT platforms) and procedural safeguards to enable cross-disciplinary collaboration; (3) Incentive and constraint mechanisms, referring to policy tools (financial/non-financial rewards, accountability metrics) designed to motivate or regulate collaborative behaviors; and (4) Structure and strength of collaborative relationships, characterized by the topology (e.g., network centrality) and resilience of inter-provider connections, measured through interaction frequency and resource-sharing patterns.,; 8 factors and 15 items whose overall reliability and validity were good and has certain applicability in China. Given regional sociocultural diversity, the findings require validation through broader case studies.

In order to cope with the changes in demand for health caused by multiple factors such as aging and rising prevalence of chronic noncommunicable diseases, different countries and regions have been exploring the construction of integrated healthcare system since the 1970s [1]. In 1987, the World Health Organization (WHO) proposed a conceptual model of a comprehensive healthcare system based on the principles of primary health [2], advocating the integration of services in disease prevention, health promotion, treatment, rehabilitation and so on, so as to promote the restructuring of the healthcare system. In 2015, the WHO issued the WHO global strategy on people-centred and integrated health services, in which it called for and guided member countries to establish and improve a people-centered and integrated healthcare system based on national realities, to ensure that citizens got access to appropriate, timely, equitable and affordable high-quality medical and health services in the most suitable places [3].

To build an integrated medical and health service means to build a fully functional health service delivery network that is interconnected with different levels and types of medical and health service providers and focuses on residents’ health, so as to provide residents with continuous health services covering the whole life expectancy, whose essence is the professional collaboration or professional integration of health technicians featuring doctors. Compared with horizontal professional collaboration, which refers to collaboration of health technicians of different specialties within the same institution or between facilities at the same level, vertical professional (collaboration of health technicians of institutions at different levels) collaboration is more difficult to implement yet is the key point to build an integrated healthcare system. Thus, it is important to develop a set of scientific and appropriate evaluation tools to comprehensively understand the status quo of vertical professional cooperation and systematically evaluate the effect of collaboration, which is conducive to timely detection of problems and better promotion of system integration. Currently, the evaluation dimensions and elements involved in the research on vertical professional cooperation evaluation tools focus on economic incentives, individual reductionism, one-dimensional structural determinism, etc. and improvements are expected in terms of comprehensiveness and integration of research perspectives, connection with the reality of developing countries [4].

As a typical representative of developing countries, China is exploring an integrated healthcare system in line with China’s reality, taking the tight county healthcare alliance as the main form of practice. Under varying socioeconomic development conditions, the challenges of professional integration manifest differently across stages. China, for example, currently faces dual imperatives: elevating the professional standing of general practitioners while addressing systemic heterogeneity in medical education. Therefore, based on “human-centered integrated healthcare system evaluation framework”, which is the theoretical premise obtained in the previous research of the research group, this study selects typical case areas of China’s close-knit county medical community practice to develop vertical professional integration evaluation tools, which not only contain more comprehensive evaluation factors and dimensions such as values and institutional factors, but connect macro factors with the group behavior of health technicians, mainly doctors, also it assess and guide the reform practice of vertical professional integration in China better while providing certain reference to other developing countries.

## Data collection and analysis methods

### Development of the questionnaire

#### Description of the evaluation framework dimensions

**Figure 1:**
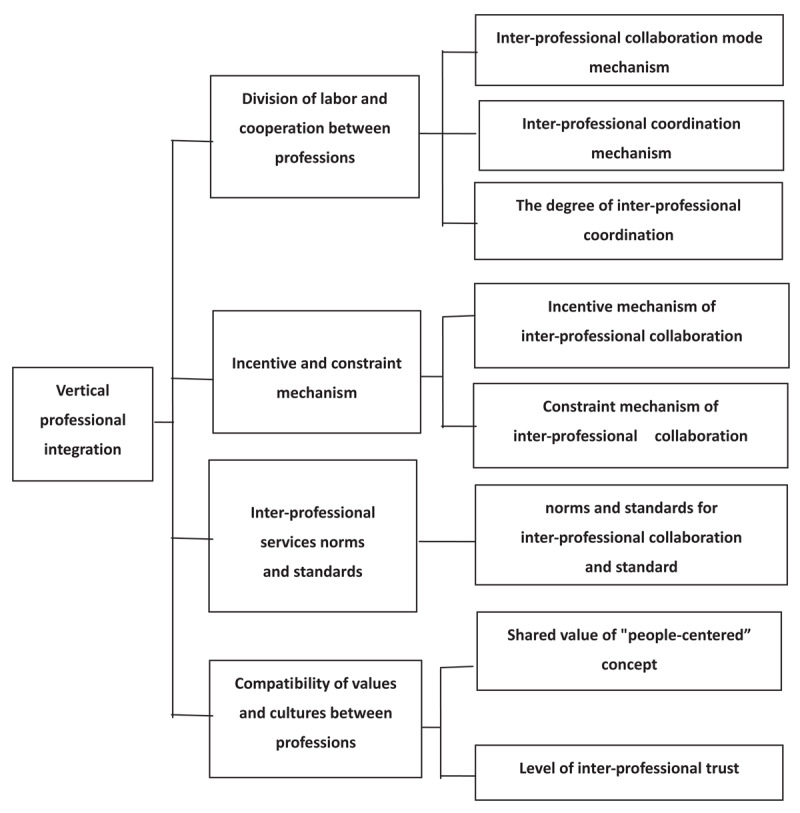
Theoretical presupposition model of dimensions and elements of vertical professional integration evaluation.

Based on the “Evaluation framework of human-centered integrated healthcare system”, the theoretical presupposition of evaluation dimensions and elements of vertical professional integration in healthcare system is designed to include 4 dimensions, each corresponds to several elements (Figure 1). Firstly, professional division of labor and cooperation refers to the division of labor and cooperation mechanism at different levels and between different professions (prevention, rehabilitation, nursing, medical treatment, etc.), such as establishment of interdisciplinary service teams, signing of cooperation agreements on the basis of projects, and the communication mechanism among professionals. It consists of three elements, namely cross-professional coordination mechanism, cross-professional cooperation mode, and the closeness of cross-professional cooperation. Secondly, the incentive and constraint mechanism refers to that between different levels and different specialties, such as the benefit distribution mechanism and the responsibility sharing mechanism among specialties. It also consists of two elements, the incentive mechanism of cross-professional cooperation and the constraint mechanism of cross-professional cooperation. Thirdly, inter-professional service norms and standards refer to the formulation of targeted, guiding and standardized inter-professional service norms and standards to guide different levels and professions to cooperate with each other based on their respective capabilities, roles, responsibilities and obligations; the key element here is inter-professional collaboration of specifications and standards. Fourthly, compatibility of cross-professional values and cultures refers to the consistency of health-related concepts, values and cultural cognition at different levels and in different professions; the two elements fall into this category are the value concept of “human-centered” shared among professionals and the degree of trust between professionals [5].

#### Establish the questionnaire entry pool and compose the questionnaire

The questionnaire entry pool is formed on the basis of the dimensions and elements of the evaluation system regarding vertical professional integration in medical health service system with reference to literature review. The research group and the expert group carried out discussions and pre-experiments to develop the evaluation questionnaire of vertical professional integration [6], deleting or modifying unsuitable items, which included 23 items (Table 1). The items were sorted and coded, considering the readability of the questionnaire, with 5-point Likert values. 1 = “Never”, 2 = “occasionally”, 3 = “usually”, 4 = “often”, 5 = “always”.

**Table 1:** Dimensions, elements, connotation and corresponding items of vertical professional integration.


DIMENSIONS	ELEMENTS AND CONNOTATIONS	ITEMS AND CODES

Division of labor and cooperation between professions	Inter-professional coordination mechanism: whether an effective inter-professional communication and coordination mechanism is established, with specialized departments or personnel organizing and coordinating multidisciplinary consultations.	7\9\10

	Inter-professional collaboration mode: whether an inter-professional collaboration mode is adopted, such as the establishment of inter-professional service team, specialist alliance, etc.	2\3

	The degree of inter-professional collaboration: How close is inter-professional coordination	1\4\5\6\8

Incentive and constraint mechanism	Incentive mechanism of inter-professional collaboration: whether relevant indicators are included into the performance appraisal, and whether the appraisal results are linked to job recruitment, individual salary, career development, etc.	12\13\14

	Constraint mechanism of inter-professional collaboration: whether there are oversight and accountability mechanisms for inter-professional collaboration.	11

Interprofessional service norms and standards	Norms and standards for inter-professional collaboration: Whether relevant norms and standards are established.	16\17\18\19

Compatibility of values and cultures between professions	Shared value of “People-centered” concept among professionals: Whether different professionals recognize “People-centered” values.	15\22\23

	Inter-professional trust: The degree of trust between different specialties.	20\21


### Data Collection

The paper-based questionnaire collected on-site was conducted among 8 township health centers December 2020, covering all 3 county-level public hospitals in D county, H Province(anonymous processing). A total of 450 doctors, nurses, laboratory technicians and other health professionals in medical and health institutions were interviewed using stratified cluster equal proportion random sampling. Altogether 450 questionnaires were distributed and 420 were effectively recovered, making the effective recovery rate 93.33% after careful examination of each copy on the spot to filter out those with wrong filling or missing items. we maintained research ethics standards by: (1) providing participants with project information sheets, (2) obtaining verbal consent, and (3) ensuring complete data anonymization.

### Analysis Method

Exploratory factor analysis: when the eigenvalue > 1 is the valid common factor, the factor load >0.35 is the meaningful item. When the correlation coefficient between the score of the item and the total score of the dimension of the item is greater than 0.2 and the correlation coefficient between the score of the item and the total score of other dimensions is less than 0.2, it is a meaningful item. Items that do not meet the above requirements can be considered for deletion as meaningless.

Reliability analysis: If the Cronbach’s α of the whole and each dimension is greater than 0.7, the reliability is good. If composite Reliability (CR) is greater than 0.7, and Average Variance extraction (AVE) is above 0.6, they can support further structural equation model analysis.

Structural equation model: P value of standardized regression coefficient being less than 0.05 indicates a statistical significance between X and Y; The model fitting index must meet the following conditions, namely Root Mean Square Error of Approximation (RMSEA) < 0.10, Comparative Fit Index (CFI) > 0.9, Normed Fit Index (NFI) > 0.9, Non-Normed Fit Index (NNFI) > 0.9. The above analysis is based on SPSS19.0.

## Research results

### General information of respondents

A total of 420 medical personnels were interviewed, including 215 males, accounting for 51.2% of the surveyed population; 234 of them were with a degree of bachelor or higher, accounting for 55.7% of the total; 159 of the interviewed were with intermediate titles, accounting for 37.9%, and 51 of them were with deputy titles and above, accounting for 10.5% of the total.

### Exploratory factor analysis

After the Bartlett spheroid test, KMO = 0.898 > 0.5, indicating that the data were suitable for exploratory factor analysis. Five common factors with eigenvalue greater than 1 and cumulative contribution rate being 72.23% were extracted with principal component and maximum variance method. Items 3, 2, 6, 7, 9, 10, 12, 13, 14, 15, 16, 17, 18, 20, 21, 22, 23 were retained because their factor loads were all greater than 0.5, and those of non-shared factors were all less than 0.5, meanwhile, the correlation coefficient of the total score of the shared factors was greater than 0.2 and that of the total score of the non-owning common factor is less than 0.2. Among them, items 18, 20, 21, 22 and 23 belong to common factor F1, items 6, 7, 9 and 10 belong to common factor F2, items 1, 13, 14 and 15 belong to common factor F3, items 2 and 3 belong to common factor F4, and items 16 and 17 belong to common factor F5 (Table 2). Item 1 and item 4 were abandoned due to their disqualified factor load of the common factor and that of the non-common factor (both were greater than 0.5) items 2, 5, 8, 11, 16 and 19 were abandoned because their correlation coefficient of the total score of the common factors was less than 0.2, and their total score coefficient of the common factors was greater than 0.2, which made them meaningless items (Table 2).

**Table 2:** Structure validity test of common factors and corresponding items.


COMMON FACTORS	ITEMS	COMMON DEGREE	FACTOR LOAD	ITEM SCORE AND TOTAL SCORE COEFFICIENT

F1	18	0.778	0.807	0.206

	20	0.778	0.832	0.244

	21	0.803	0.819	0.216

	22	0.825	0.856	0.240

	23	0.793	0.824	0.245

F2	6	0.715	0.769	0.239

	7	0.728	0.754	0.230

	9	0.834	0.811	0.249

	10	0.695	0.747	0.254

F3	12	0.707	0.765	0.264

	13	0.765	0.868	0.339

	14	0.778	0.879	0.351

	15	0.535	0.672	0.272

F4	2	0.671	0.452	0.789

	3	0.654	0.719	0.388

F5	16	0.820	0.533	0.786

	17	0.804	0.814	0.560


### Reliability analysis

The model had good convergence, which supported further structural equation model analysis. The overall reliability was good, with Cronbach’s α = 0.846. The reliability of all dimensions was generally good, and the Cronbach’s coefficients of common factors F1, F2, F3, F4 and F5 were 0.929, 0.893, 0.822, 0.643, 0.81, respectively, and the Cronbach’s α of the other common factors were greater than 0.7 except F4. The component reliability (CR) of common factors F1, F2, F3, F4 and F5 were all greater than 0.7, being 0.920, 0.880, 0.880, 0.818 and 0.787 and the average coefficient of variation extraction (AVE) were all greater than 0.6, being 0.697, 0.647, 0.650, 0.693 and 0.648.

### Structural equation model

In the initial model (M0), of F1, F2, F3 and F4 were retained for their P values of the standard regression coefficients of the degree of professional integration were all less than 0.05. Items 18, 20, 21, 22 and 23 were retained for their corresponding P values of F1 standard regression coefficients were all less than 0.05. Items 6, 7, 9 and 10 were retained for their corresponding P values of the standard regression coefficients of F2 were all less than 0.05. Items 12, 13, 14 and 15 were retained for their corresponding P values of the standard regression coefficients were all less than 0.05. And items 2 and 3 were retained for their corresponding P values of the standard regression coefficients of F4 were all less than 0.05. of Meanwhile, F5 (corresponding items 16 and 17) was abandoned because its P value of the standard regression coefficient was greater than 0.05, indicating no impact on the degree of professional integration.

In the revised model (M1), F1, F2, F3 and F4 were retained because their P values of the standard regression coefficients for the degree of professional integration were all less than 0.05, and those of the corresponding items and factors were all less than 0.05. The model fitting indexes of the revised model (M1) were all better than those of the initial model (M0) (Tables 3 and 4).

**Table 3:** Model fitting index.


	RMSEA	CFI	NFI	NNFI

Standard	<0.10	>0.9	>0.9	>0.9

The initial model (M0)	0.098	0.905	0.883	0.881

The revised model (M1)	0.097	0.922	0.903	0.9


**Table 4:** Evaluation dimensions, elements, and their corresponding items.


COMMON FACTORS	DIMENSIONS	ELEMENTS	ITEMS

F1	Inter-professional service norms and standards	Specifications and standards for Inter-professional collaboration	18. Do you think the current service specifications and standards for inter-professional collaboration are reasonable?

	Compatibility of values and cultures between professions	Degree of trust between different specialties.	20. Do you think inter-professional collaboration is helpful to improve the quality of medical services in county-level hospitals?

			21. Do you think inter-professional collaboration is helpful to improve the quality of medical services in primary medical institutions?

		Sharing “People-centered” values among professionals	22. Do you think inter-professional collaboration is helpful in providing integrated and continuous medical and health services to patients?

			23. Do you think it is necessary to carry out inter-professional collaboration?

F2	Division of labor and cooperation between professions	Closeness of inter-professional collaboration.	6. Do you refer to diagnoses and treatment plans of other medical institutions in the medical community?

		Inter-professional coordination mechanism	7. Is specific coordination available for you to have inter-professional collaboration with other doctors?

			9. Have you ever conducted collaboration with doctors from other medical institutions in the medical community on the medical information platform?

			10. Have you ever obtained patient’s previous diagnosis and treatment information from the medical information platform?

F3	Incentive and constraint mechanism	Incentive mechanism for inter-professional collaboration	12. Does engagement in inter-professional collaboration add up to your performance appraisal score?

			13. Does engagement in inter-professional collaboration increase your income?

			14. Does engagement in inter-professional collaboration boost your career development such as professional title promotion?

	Compatibility of values and cultures between professions	Sharing “People-centered” values among professionals	15. Does engagement in inter-professional collaboration have a positive impact on trust between you and your patients?

F4	Division of labor and cooperation between professions	Inter-professional coordination mechanism	2. What professional collaborations have you participated in the medical community?

			3. What information do you have to contact doctors in other medical institutions within the community?


## Discussion

### The dimension of “value compatibility and trust” was essentially to measure the degree of the consensus of medical and health service providers at different levels on collaborative relationships

The common factor F1 was renamed as “value compatibility and trust”, referring to the consistency of health-related values, culture and behavior norms of between different levels and different professional medical and health service providers, including the two elements of value compatibility and trust, which in essence reflected the degree of consensus reached by different levels and professional medical and health service providers on the subjective meaning of vertical professional integration as a social action, which was conducive to the formation of the legitimate order of collaboration [7].

First, value compatibility was an important aspect of value integration. This element was originally named “subjective norm compatibility”, whose essence was the subjective cognition and consensus of different action subjects to the rules, and was a sociological order of tradition and habit. Value compatibility can form moral constraints, so that individuals perceive social environment pressure when deciding whether to perform a specific behavior, reflect the influence of important others or groups on individual behavior and are influenced by normative beliefs and obedience motives, and moral constraints formed by value compatibility are considered to be important factors affecting doctors’ autonomous professional behavior [8,9]. Item 18 measured perception of rationality of existing norms and standards of interprofessional collaboration by different professional medical and health service providers, which originally belonged to the dimension of “norms and standards of interprofessional collaboration”, but was eventually included in the dimension of “value compatibility”, indicating that the perception of normative rationality was part of the degree of value integration. This, similar to the recognition of the “human-centered” value concept measured with items 22 and 23, fell into the category of “value compatibility”, while showing consistency with the results of relevant studies on doctors’ referral behavior, indicating doctors’ referral behavior being altruistic [10].

Next, the degree of interprofessional trust. Trust is the basis of cooperative behavior. In a broad sense, trust refers to a belief or state of mind in which the donor believes that the fiduciary will do its best to fulfill its commitments and will not exploit the weakness of the donor to seek improper benefits. In the previous theoretical presupposition, the trust relationship between medical and health service providers of different levels and specialties was also regarded as an important element in the concept and value dimension that affected vertical professional integration [11]. From the empirical results of this study, items 20 and 21 measured the degree of trust between different levels and different professions, and further indicated that there was a direct correlation between the degree of trust and vertical professional cooperation behavior, and the possibility of vertical professional integration behavior can be speculated by measuring the degree of trust relationship between different levels and different professions, which was consistent with the previous theoretical presupposition.

### “Communication and coordination mechanism” was an important prerequisite for the cooperation between different parities whose essence is the flow of information elements and its guarantee mechanism

Common factor F2 was renamed as “communication and coordination mechanism” to reflect the flow of information elements and its guarantee mechanism for collaboration between medical and health service providers at different levels and indifferent areas of expertise, including two elements of health information support system and organizational guarantee.

Health information support system was supposed to be interconnected Item 6 “Do you refer to diagnoses and treatment plans of other medical institutions in the medical community?”, item 9 “Have you ever conducted collaboration with doctors from other medical institutions in the medical community on the medical information platform?”, and item 10 “Have you ever obtained patient’s previous diagnosis and treatment information from the medical information platform?” evaluated and measured the interconnection of information through the use of health information system by medical staff, which further explained that the interconnection of health information system was an important factor to promote vertical professional integration. This was similar to some previous study results, which adopted the theory of planned behavior and proposed that information interconnection had a positive influence on perceptual behavior control, and indirectly promoted two-way referral intention through perceptual behavioral control [12,13].

The second element was about organizational guarantee mechanism. Item 7 “Is specific coordination available for you to have inter-professional collaboration with other doctors?” manifested requirement for special personnel to be responsible for inter-professional coordination. This is consistent with the classical theoretical model of professional collaboration, Chronic Care Model (CCM), which clearly stated that dedicated coordination was an important organizational guarantee element, meaning “designating a medical service worker as a case manager to improve the coordination and continuity of the service system” [14,15]. At the same time, in the specific practice at home and abroad, the establishment of a special person responsible for the coordination of different medical institutions to ensure the realization of two-way referral is also widely emphasized. For example, in the management of chronic diseases in Germany, a case manager is designated to help build collaboration between different professional medical and health service providers [16]. In practice in China, the Shanghai hierarchical diagnosis and treatment plan clearly requires that “each medical institution within the medical consortium set up a coordination department for referral, and assign special personnel be responsible for the work contact between the institution and the upper and lower medical institutions of the medical consortium and the coordination between departments within the institution [4,17].

### “Incentive and constraint mechanism” included material and non-material factors that affect vertical professional integration behavior

Common factor F3, named “incentive and constraint mechanism” worked as a mechanism to guide and motivate professional cooperation behavior with incentives that influenced medical staff, involving performance appraisal and income, professional title promotion, doctor-patient trust and other aspects, both material and non-material, which was a core dimension to promote vertical interprofessional collaboration [1,18].

Firstly, economic income and career development are common components of incentive and constraint mechanisms, focusing on material and economic incentives. This is consistent with the presupposition of the previous theoretical framework. that is, material and economic factors such as economic income and career promotion space play an important role in stimulating and guiding the vertical professional integration behavior of different levels and different professional health service providers [19]. Most of the studies have focused on the influence of economic incentives on the behavior of doctors, emphasizing the use of rational and purposeful policy tools such as medical insurance payment, salary and administrative power to play the role of formal institutions in shaping doctors’ group behavior [20].

Secondly, doctor-patient trust is a new component of incentive and restraint mechanism which belongs to interpersonal capital and is a non-material incentive factor. Item 15 “Does engagement in inter-professional collaboration have an positive impact on trust between you and your patients?” measured patient-trust relationship, which previously belonged to the “sharing of people-centered values among professionals” and later incorporated into the “incentive and constraint mechanism”, supplementing the role of previously overlooked non-material incentives. Some existing research results also showed that the doctor-patient trust relationship, as an interpersonal capital in the doctor-patient social relationship, had a positive incentive and promotion effect on improving the doctor’s referral behavior [21]. At the same time, the doctor-patient trust relationship manifested interpersonal capital status between doctors and patients, rather than that between medical and health service providers of different levels and professions. Therefore, compared with the dimension of “shared value concept among professions”, the dimension of doctor-patient trust was more in line with the dimension of “incentive and constraint mechanism” logically, whose content validity was better [22].

### The “structure and strength of cooperation relationship” reflected the strength of social network structure and connection between health technicians at different levels and in different professions

The common factor F4 was renamed as “structure and strength of cooperation relationship”, referring to the structure and strength of the social networks among medical and health service providers at different levels and with different specialties, including two elements: cooperative relationship structure and cooperative relationship strength.

The essence of the division of labor and cooperation mechanism is the position, function and cooperation mode of different levels and types of medical and health service providers in the collaborative network, which reflects the structure and intensity of the collaborative relationship. Specifically, the first is the structure of cooperative relationship. Item 2 “What business collaborations have you participated in in the medical community?” originally belonged to “division of labor and cooperation mode” in the dimension of “division of labor and cooperation mechanism” and indicated the positioning and cooperation mode of different levels and professional medical and health service providers in the collaboration network, which was in line with the definition of social network structure, because of what it was named the collaborative relationship structure. This coincided with results of some studies, among which formal collaborative relationships such as referral – training, referral – support, chronic disease – telemedicine, information – organization management, friendship – referral, friendship – telemedicine, friendship – chronic disease, friendship – support were pointed out to have positively affected each other and have been helped with informal friendship networks in formation. Social network structure promoted vertical professional integration [23]. The second is the strength of the collaborative relationship. What came next was the strength of collaborative relationship. Item 3 “What information do you have to contact doctors in other medical institutions within the community?” measured the frequency and closeness of the contact between health technicians, mainly doctors, reflecting the strength of collaborative relationships. This was consistent with the results of previous studies, which pointed out that the stronger the social network connection, the higher the closeness and frequency of connection, which could promote the trust relationship between collaborators, and then positively affected the ability of knowledge integration, including systematization, socialization and collaboration [24].

## Conclusion

**Figure 2:**
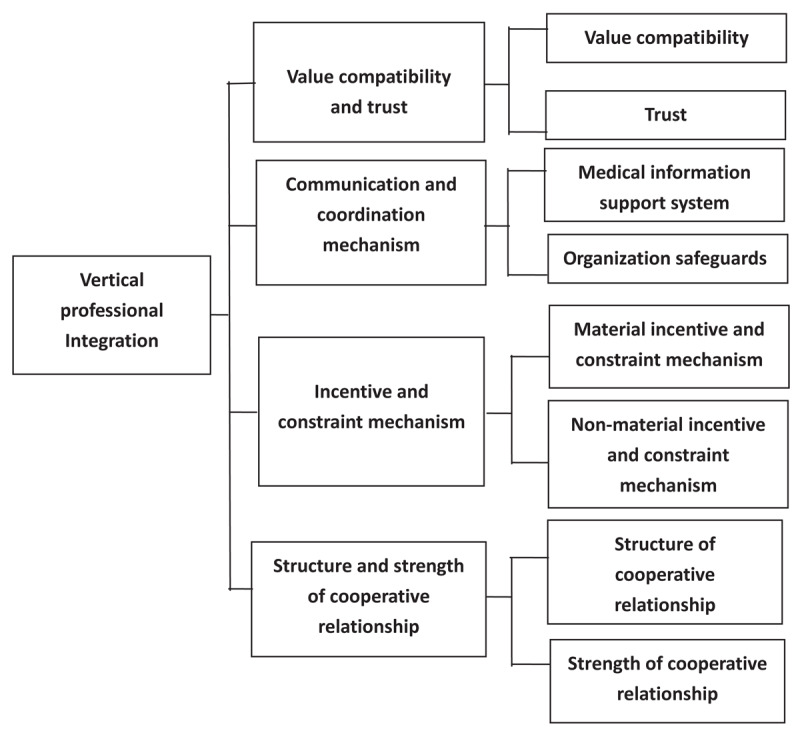
Verified theoretical model of the evaluation dimensions and elements of vertical inter-professional collaboration of healthcare system.

The measurement tool of vertical professional collaborative evaluation tools of healthcare system proposed in this study contained 4 dimensions, 8 factors, and 15 measurement items. Among them, the dimension of “value compatibility and trust” corresponded to two factors and five measurement items: value compatibility (item 18/22/23) and trust (item 20/21); The dimension of “communication and coordination mechanism” corresponded to 2 factors and 4 measurement items: health information support system (item 6/9/10), organizational safeguards (item 7); The dimension of “incentive and constraint mechanism”, corresponded to 2 factors and 4 measurement items: material incentive and constraint mechanism (item 12/13/14), non-material incentive and constraint mechanism (item 15); The dimension of “Structure and strength of cooperative relationship” corresponded to two factors and two measurement items: structure of cooperative relationship (item 2) and strength of cooperative relationship (item 3). The overall reliability and validity of this evaluation tool were good, which applied to the vertical professional cooperation evaluation of healthcare system in China to certain extend, and was supposedly to provide reference to other developing countries. At the same time, this study had certain limitations. Due to the complex connotation and measurement of evaluation dimensions and factors involved in vertical professional integration, the empirical research was confined in D county of H Province, therefore further extrapolation is needed Meanwhile, evaluation dimensions and factors are expected to be more profoundly explored and empirical researches are supposed to be done in more diverse regions to better adjust and validate the evaluation tool (Figure 2 and Table 5). This study was conducted solely in D County. Given regional sociocultural diversity, the findings require validation through broader case studies.

**Table 5:** Verified evaluation dimensions, elements and corresponding items of vertical professional integration in healthcare system.


COMMON FACTORS	DIMENSIONS	ELEMENTS	ITEMS AND CODES

F1	Value compatibility and trust Definition: The alignment of health-related values, cultural norms, and behavioral expectations across vertically tiered (e.g., primary vs. tertiary care) and horizontally distinct (e.g., physicians vs. nurses) healthcare providers.	Value compatibility	18. Do you think the current service specifications and standards for professional collaboration are reasonable?

			22. Do you think professional collaboration is helpful in providing integrated and continuous medical and health services to patients?

			23. How do you see the need for professional collaboration?

		Trust	20. Do you think professional collaboration is helpful to improve the quality of medical services in county-level hospitals?

			21. Do you think professional collaboration is helpful to improve the quality of medical services in primary medical institutions?

F2	Division of professional labor and cooperation Definition: Systems governing the bidirectional flow of clinical/administrative information and procedural safeguards to enable cross-level, cross-specialty collaboration.	Information system	6. Do you refer to diagnoses and treatment plans of other medical institutions in the medical community?

			9. Have you collaborated with doctors from other medical institutions in the medical community on the medical information platform?

			10. Have you ever obtained the patient’s previous diagnosis and treatment information from the medical information platform?

		Organization safeguards	7. Is there someone to coordinate your collaboration with your collaborating doctors?

F3	Incentive and constraint mechanism Definition: Policy-driven tools (financial/non-financial) designed to promote or deter specific collaborative behaviors among healthcare professionals.	Material incentive and constraint mechanism	12. Does engagement in inter-professional collaboration add up to your performance appraisal score?

			13. Does engagement in inter-professional collaboration increase your performance earnings?

			14. Does engagement in inter-professional collaboration boost your career development such as professional title promotion?

		Non-material incentive and constraint mechanism	15. Does engagement in inter-professional collaboration have a positive impact on trust between you and your patients?

F4	Structure and strength of cooperative relationship Definition: The topology (e.g., centrality, density) and resilience of social/professional networks linking multi-level, multi-disciplinary providers.	Structure of cooperative relationship	2. What professional collaborations have you participated in the medical community?

		Strength of cooperative relationship	3. What information do you have to contact doctors in other medical institutions within the community?


## References

[1] Hu J, Zheng Y, Dai T, Wang QB, Li L. Core elements and development characteristics of the theoretical framework of integrated healthcare system: a systematic review. Chinese Journal of Health Policy. 2022;15(1):11–19.

[2] World Health Organization. Hospitals and health for all: report of a WHO Expert Committee on the Role of Hospitals at the First Referral Level. Geneva: World Health Organization; 1987. 3107221

[3] World Bank Group, World Health Organization, Ministry of Finance of the People’s Republic of China, et al. Deepening China’s health system reform: a policy framework for establishing value-based high-quality healthcare delivery. Beijing: World Bank; 2016.

[4] Li YH. Study on the Evaluation of Professional Collaboration on Health Care Alliance in Tianchang City of Anhui Province [dissertation]. Beijing: Peking Union Medical College; 2020.

[5] Chen ZR. Study on evaluation of framework of integrated health care system [dissertation]. Beijing: Peking Union Medical College; 2018.

[6] Li YH, Dai T, Zheng Y, et al. Factors influencing professional collaboration among county- and township-level healthcare workers in Tianchang City, Anhui Province. China Medical Herald. 2020;17(14):52–56. DOI: 10.20047/j.issn1673-7210.2020.14.013

[7] Weber M. The basic concepts of sociology; economic action and social groups. Translated by GU ZH, KANG L, JIAN HM. Shanghai: Shanghai Joint Publishing; 2020:56–66.

[8] Noar SM. Health behavior theory and cumulative knowledge regarding health behaviors: are we moving in the right direction? Health Educ Res. 2005;20(3):275–290. DOI: 10.1093/her/cyg113 15632099

[9] Yao ZL. Between interest and morality: a sociological analysis of professional autonomy of urban doctor in contemporary China. Beijing: China Social Sciences Press; 2017.

[10] Huang J, Wang PL, Guo K, et al. Analysis of two-way referral behavior of doctors in medical alliance based on the theory of planned behavior. Chin Hosp Manag. 2019;39(6):35–37.

[11] Ruan LN. Research on coordination mechanism of urban community health service network [dissertation]. Nanchang: Nanchang University; 2012.

[12] Zhang Y. A study on the influence of information interconnection on doctors’ two-way referral intention-based on theoretical of planned behavior [dissertation]. Beijing: Peking Union Medical College; 2023.

[13] Zhang Y, Huang J, Dai T. Development of a qualitative model explaining the association of informatization with physicians’ intentions and behaviors related to bi-directional referrals. Chin Gen Pract. 2022;25(13):1636–1641.

[14] Wagner EH, Davis C, Schaefer J, et al. A survey of leading chronic disease management programs: are they consistent with the literature? Manag Care Q. 1999;7(3):56–66. 10620960

[15] Zheng Y, Hu J, Li L, Dai T. Practice and enlightenment of chronic disease management at the county level in China from the perspective of professional integration: a qualitative case study of Youxi County, Fujian Province. Int J Integr Care. 2023;23(3):6. DOI: 10.5334/ijic.7550 PMC10417912 37577141

[16] Yu HX, Feng YM, Fu M, et al. International experience on the division and cooperation among medical institutions and its implications for China: an analysis of the UK, Germany, Singapore and the US. Chin J Health Policy. 2014;7(6):10–15.

[17] Hu B. Overall idea, key areas and implementation on path of accelerating the improvement of the healthcare system in Shanghai. Sci Dev. 2023;(174):97–105.

[18] Li F, Bai X, Chen D, et al. Thoughts on the connotation and key measures of integrating health services. Health Econ Res. 2019;36(3):9–12.

[19] Zhang MX. Study on management model of two-way referral operations for community health services institutions and hospital [dissertation]. Wuhan: Huazhong University of Science and Technology; 2009.

[20] Ma Q, Zou XM, Huang T, et al. Analysis on incentive compatibility system of medical alliance in medical collaboration model. Soft Sci Health. 2021;35(1):35–38. DOI: 10.12816/0047385

[21] Li L, Zheng Y, Zhu XL, Hu J. Research on the establishment and suitability of an assessment tool for demand-based integration of county-level health services in China. Chin J Health Policy. 2022;15(1):29–36.

[22] Guo SL. Study on incentive and restraint mechanism of dual referral between urban hospital and community health service institutions [dissertation]. Wuhan: Huazhong University of Science and Technology; 2008.

[23] Tan M. Research on the organization characteristics of county health services community and effect perception from the perspective of social network [dissertation]. Wuhan: Huazhong University of Science and Technology; 2021.

[24] Zhu L. Research on the relationship between social network connection strength, knowledge integration ability and employees’ innovation performance [dissertation]. Zhenjiang: Jiangsu University; 2019.

